# MFGE8 Does Not Influence Chorio-Retinal Homeostasis or Choroidal Neovascularization *in vivo*

**DOI:** 10.1371/journal.pone.0033244

**Published:** 2012-03-15

**Authors:** William Raoul, Lucie Poupel, David-Alexandre Tregouet, Sophie Lavalette, Serge Camelo, Nicole Keller, Sophie Krumeich, Bertrand Calippe, Xavier Guillonneau, Francine Behar-Cohen, Salomon-Yves Cohen, Holger Baatz, Christophe Combadière, Clotilde Théry, Florian Sennlaub

**Affiliations:** 1 INSERM, U968, Paris, France; 2 University Pierre and Marie Curie, Institut de la Vision, Paris, France; 3 Centre National de la Recherche Scientifique, Paris, France; 4 Assistance Publique-Hôpitaux de Paris, Hôtel Dieu, Service d'Ophtalmologie, Paris, France; 5 INSERM, UMR S945, Laboratory of Immunity and Infection, Paris, France; 6 University Pierre and Marie Curie, Laboratory of Immunity and Infection, Paris, France; 7 Assistance Publique-Hôpitaux de Paris, Groupe Hospitalier Pitié-Salpétrière, Service d'Immunologie, Paris, France; 8 INSERM, UMR S 872, Centre de Recherche des Cordeliers, Paris, France; 9 University Pierre and Marie Curie, Paris, France; 10 Université Paris Descartes, Paris, France; 11 INSERM, U932, Paris, France; 12 Institut Curie, Centre de Recherche, Paris, France; 13 Augenärztliche Gemeinschaftspraxis, Augenzentrum Recklinghausen und Zentrum der Augenheilkunde, J-W Goethe Universität Frankfurt am Main, Frankfurt, Germany; 14 Centre d'Angiographie et de Laser, Paris, France; 15 INSERM, UMR S 937, Faculté de Médecine La Pitié-Salpêtrière, Paris, France; University of Florida, United States of America

## Abstract

**Purpose:**

Milk fat globule-epidermal growth factor-factor VIII (MFGE8) is necessary for diurnal outer segment phagocytosis and promotes VEGF-dependent neovascularization. The prevalence of two single nucleotide polymorphisms (SNP) in *MFGE8* was studied in two exsudative or “wet” Age-related Macular Degeneration (AMD) groups and two corresponding control groups. We studied the effect of MFGE8 deficiency on retinal homeostasis with age and on choroidal neovascularization (CNV) in mice.

**Methods:**

The distribution of the SNP (rs4945 and rs1878326) of *MFGE8* was analyzed in two groups of patients with “wet” AMD and their age-matched controls from Germany and France. MFGE8-expressing cells were identified in *Mfge8* ^+/−^ mice expressing ß-galactosidase. Aged *Mfge8* ^+/−^ and *Mfge8* ^−/−^ mice were studied by funduscopy, histology, electron microscopy, scanning electron microscopy of vascular corrosion casts of the choroid, and after laser-induced CNV.

**Results:**

rs1878326 was associated with AMD in the French and German group. The *Mfge8* promoter is highly active in photoreceptors but not in retinal pigment epithelium cells. *Mfge8^−/−^* mice did not differ from controls in terms of fundus appearance, photoreceptor cell layers, choroidal architecture or laser-induced CNV. In contrast, the Bruch's membrane (BM) was slightly but significantly thicker in *Mfge8^−/−^* mice as compared to controls.

**Conclusions:**

Despite a reproducible minor increase of rs1878326 in AMD patients and a very modest increase in BM in *Mfge8^−/−^* mice, our data suggests that MFGE8 dysfunction does not play a critical role in the pathogenesis of AMD.

## Introduction

Milk fat globule-EGF-factor (MFGE8), also named lactadherin, PAS 6/7, SED1, BA46, p47, is a secreted glycoprotein first described in milk fat globules released in milk by mammary epithelial cells [1], [2]. Secreted by different cell types, it promotes phagocytosis by linking phosphatidylserine at the surface of membrane vesicles [3] and apoptotic cells [4] to the αvβ3/β5 integrin on phagocytic cells. MFGE8 mediated phagocytosis induces a regulatory T cell response [5] and *Mfge8^−/−^* mice develop spontaneous late onset lupus-like disease and glomerulonephritis [6]. In humans, the association of two nucleotide polymorphisms in the coding region of *MFGE8* predisposes subjects to systemic lupus erythematosus [7], suggesting that these single nucleotide polymorphisms (SNP) lead to a dysfunctional MFGE8. Furthermore, MFGE8 binds to αvβ3/β5 of vascular endothelial cells and promotes VEGF-driven neovascularization [8].

Phagocytosis of spent outer segments (OS) is critical for the long-term maintenance of the retina [9], [10] and dependent upon a tyrosine kinase receptor (Mertk) [11], [12] and the αvβ5 integrin [13]. The neural retina and the retinal pigment epithelium (RPE) express MFGE8 [14], whose interaction with αvβ5 integrin of RPE cells is essential in the diurnal OS phagocytosis [15]. Furthermore, we have previously shown that OS phagocytosis by the RPE is necessary for choroidal maintenance *in vivo* [16].

Age-related macular degeneration (AMD) is a major cause of central vision loss in the elderly in Western countries [17]. AMD's most prominent pathological features are lesions involving the RPE and Bruch's membrane (BM), the degeneration of photoreceptors, [18] and VEGF-driven choroidal neovascularization (CNV) [19], that occurs approximately in 10% of patients. The causes of AMD are not well understood, but epidemiological studies [20] and murine models [21] have identified key factors in its pathogenesis such as age [17], [22], family history [23] and smoking [24], [25].

In summary, MFGE8 has been shown to be essential in diurnal OS phagocytosis and in angiogenesis. In the eye, its dysfunction could therefore lead both to retinal degeneration and choroidal involution as a consequence of impaired RPE phagocytosis and/or to inhibition of neovascularization. We investigated whether a SNP associated with increased prevalence of lupus in human patients [7] is associated with AMD. Furthermore, we investigated whether the deficiency of MFGE8 affects chorioretinal homeostasis and CNV *in vivo* using *Mfge8^−/−^* mice.

## Materials and Methods

### Ethics statement

In accordance with the Declaration of Helsinki, patients and volunteers provided written and informed consent for the studies, which were approved by the Hôtel Dieu ethics committee (CCPPRB no. 0611303) and by the ethics committee of the Ärztekammer Westfalen-Lippe and the University of Münster (no. 2007-246-f-S).

Animal experiments were approved by the Institutional Animal Care and Use Committee, “Comité d'éthique pour l'expérimentation animale Charles Darwin” (ID Ce5/2010/002), and treated in compliance with the ARVO Statement for the Use of Animals in Ophthalmic and Vision Research.

### Single Nucleotide Polymorphisms analysis in AMD

Studies, including participants' assessments and ethics details of each cohort were described in Combadiere *et al.* [26] and Baatz *et al.* [27]. White Caucasian control subjects and AMD patients were recruited in Paris (France: 251 control subjects, 274 “wet” AMD patients) and in Recklinghausen (Germany: 317 control subjects, 263 “wet” AMD patients). The mean age and gender distribution are summarized in Tables 1 and 2.

**Table 1:** The mean age and gender distribution in the patient and control groups.

Patients	FCTL, n (%)	FAMD, n (%)
Men	91 (36,25)	81 (29,56)
Women	160 (63,75)	193 (70,44)
Total	251	274
Mean Age	71,8+/−7,75	79,5+/−6,72
*P* value (χ^2^ test)	0.1	

French groups (control group : FCTL; AMD group : FAMD).

**Table 2:** The mean age and gender distribution in the patient and control groups.

Patients	GCTL, n (%)	GAMD, n (%)
Men	115 (43,73)	126 (39,75)
Women	148 (56,27)	191 (60,25)
Total	263	317
Mean Age	77,8+/−5,38	79,2+/−5,47
*P* value (χ^2^ test)	0.3	

German groups (control group : GCTL; AMD group : GAMD).

Rs4945 is in open reading frame SNP and leads to Arginine replacement of a Serine in the amino acid number 3. Rs1878326 is also in open reading frame SNP of *MFGE8* and leads to Leucine replacement of Methionine in the amino acid number 76. Allelic frequencies were calculated by gene counting.

We used the chi-square test (χ^2)^ to compare genotype distributions and allele frequencies in participants. The Hardy-Weinberg equilibrium was tested using a χ^2^ test with 1 degree of freedom. Association between SNP and “wet” AMD was assessed by use of the Cochran-Armitage's trend test [28]. Linkage disequilibrium and haplotype association analyses were performed by the use of THESIAS software [29].

### Animals

*Mfge8^−/−^* and *^+/−^* mouse strains were generated as described before [8]. *Mfge8^−/−^* are functionally deficient in MFGE8 due to a gene-trap insertion of ß-galactosidase in the *Mfge8* gene (C57Bl/6 background, N8). The mice were maintained at the Institut Curie animal facility or Centre de Recherche des Cordeliers animal facility (Paris, France) under pathogen-free conditions. All animals were housed in a 12/12 hour light/dark (100–500 lux) cycle with food and water available *ad libitum*.

### Fundus Photography and Laser-Photocoagulation

Mice were anesthetized by intraperitoneal injection of pentobarbital (40 mg/kg). Pupils were fully dilated with 1% tropicamide. Coverslips positioned on the mouse cornea were used as a contact glass. Fundus photographs were taken with a digital CCD camera (Nikon D3) coupled with an endoscope (Karl Storz, Guyancourt, France) as previously described [30].

Laser-photocoagulations were performed 1 to 2 disc diameters away from the papillae with an Argon laser (Viridis 532 nm, Quantel Medical, Clermont-Ferrand, France) mounted on a slit lamp (400 mW, 50 ms and 50 µm; Hagg-Streitt, BQ 900). For CNV visualization at day 14, 3 months old mice were anesthetized and perfused through their heart with phosphate buffered saline (PBS) containing fluorescein (FITC)-dextran 50 mg/ml (2.10^6^ mW). Animals were sacrificed with 100% CO_2_ and their eyes were removed and processed as described below. CNV was quantified on photographs with Image J analysis software. CNV was quantified as FITC positive surface.

### Choroidal Flatmounts and Immunohistochemistry

The eyes were enucleated, fixed in 4% paraformaldehyde (PFA) for 20 minutes at room temperature, and sectioned at the limbus; the cornea and lens were discarded. The retinas were peeled from the RPE/choroid/sclera. Retinas and choroids were incubated with the indicated primary and secondary antibodies. The choroids and retinas were radially incised and flatmounted. The primary antibodies used were rabbit anti-IBA1 (1∶400; Wako, Neuss, Germany) and rhodamine phalloidine (1∶200; Invitrogen, Cergy-Pontoise, France). Sections were counterstained with 4–6-diamino-2-phenylindole (DAPI). Choroids, retinas and sections were viewed with a fluorescence microscope (DM5500B, Leica, Nanterre, France).

For ß-galactosidase activity detection, frozen sections from eyes were fixed in 4% PFA before incubation. The samples were then incubated in the beta-galactosidase detection reagent (5 mM potassium ferricyanide, 5 mM potassium ferrocyanide, 2 mM MgCl, 0.30 mg X-gal/ml in PBS) overnight at 37°C before mounting in Immumount (Thermo Scientific, Courtaboeuf, France).

### Histology and Electron Microscopy

For histology, eyes were fixed in 0.5% glutaraldehyde/4% PFA for 2 h, dehydrated and mounted in historesin. 5-µm sections were cut and stained with toluidin blue. For electron microscopy, eyes were fixed in 2.5% glutaraldehyde of cacodylate buffer (0.1 M, pH 7.4). After 1 hour, eyeballs were dissected, fixed for another 3 hours, post-fixed in 1% osmium tetroxide in cacodylate buffer, and dehydrated in graduated ethanol solution. The samples were included in epoxy resin and oriented. Semi-thin sections (1 µm), obtained with an ultramicrotome Reichert Ultracut E (Leica), were stained with toluidin blue, examined with a light microscope, and photoreceptor layer thickness was measured. Ultra-thin sections (80 nm) were contrasted by uranyl acetate and lead citrate, then observed in an electron microscope JEOL 100 CX II (JEOL, Tokyo, Japan) with 80 kV.

### Vascular Corrosion Casts

Animals were sacrificed by CO_2_ inhalation. Vascular corrosion cast was performed as previously described by Houssier *et al.* [16]. Briefly, a perfusion was done with Mercox resin+catalyst (Ladd Research/Inland, Saint Loup sur Semouse, France) into the aorta through left heart ventricle. After removal, eye tissues were conserved overnight at 37°C in PBS to allow complete polymerisation and digested by 5% KOH during 2 weeks at 37°C until only the vascular corrosion casts remained. The specimens were prepared for electron microscopy, then scanned and analyzed using Image J Software (NIH). The avascular area was measured on frontal views and expressed as the percentage of intercapillary surface of the whole area. The thickness of choriocapillaries was measured on perpendicular views of the cast from the retinal to scleral side of the choriocapillary cast.

### Reverse Transcription and Real Time Polymerase Chain Reaction

After tissues were separated (neural retina and choroid/RPE), total RNA was isolated with NucleoSpin RNA II Kit (Macherey-Nagel, Hoerdt, France). Single-stranded cDNA was synthesized from total RNA (pre-treated with DNaseI amplification grade) using oligodT as primer and superscript reverse transcriptase (Invitrogen, Cergy Pontoise, France). Subsequent real-time polymerase chain reaction (RT PCR) was performed using cDNA, qPCR SuperMix-UDG Platinum SYBR Green (Invitrogen), and the following primers (0.5 pmol/µl): mm actin sense: 5′-AAGGCCAACCGTGAAAAGAT-3′; mm actin antisense : 5′- GTGGTACGACCAGAGGCATAC-3′; mm *Mfge8* sense : 5′-GGATAATCAGGGCAAGATCA-3′; mm *Mfge8* antisense : 5′-TAGGACGCCACATACTGGAT-3′

PCR reactions were performed in 40 cycles of 15 s at 95°C, 45 s at 60°C. Product was not generated in control reactions in which reverse transcriptase was omitted during cDNA synthesis.

### Statistical Analysis

Graph Pad Prism 5 (GraphPad Software, San Diego, CA, USA) was used for the data analysis and graphic representations. All values are reported as means ± SEM. Statistical analysis was performed using the Mann-Whitney test for comparison among means between two groups or ANOVA test between more than two groups. Significance was set at *P*<0.05.

## Results

### *MFGE8* single nucleotide polymorphism studies in two groups of wet AMD patients and their controls

Two common SNP were previously identified in the open reading frame of the *MFGE8* gene called *MFGE8* R3S (rs4945 for nucleotide C>A) and *MFGE8* M76L (rs1878326 for nucleotide 226 A>C), associated with an increased risk of systemic lupus erythematosus [7]. The fact that *Mfge8^−/−^* mice develop a spontaneous late onset lupus-like disease and glomerulonephritis [6] suggests that rs4945 and rs1878326 lead to a decrease of MFGE8 bioactivity, that would explain their association with the human disease.

We thus investigated the described *MFGE8* SNP in two DNA collections of “wet” AMD patients recruited in France (FAMD, 274 subjects) and in Germany (GAMD, 317 subjects) and their age matched controls (FCTL, 251 subjects; GCTL, 263 subjects). The mean age and gender distribution are summarized in Tables 1 and 2. All of the subjects were of caucasian origin or decent. Risk factors for “wet” AMD such as Factor H and smoking were more frequent among cases than among controls as previously described [31].

Genotype frequencies distribution of the rs4945 C>A (R3S) and rs1878326 A>C (M76L) are shown in Tables 3 and 4, respectively. All genotype distributions were compatible with Hardy-Weinberg equilibrium (*P*>0.05) in cases and in controls, from both French and German samples. In the French sample, the rs4945 -A allele was more frequent in cases than in controls (0.36 vs 0.29, *P* = 0.017) but the inverse trend was observed in German (0.30 vs 0.33, *P* = 0.229). Conversely, the pattern of association of the rs1878326 with AMD was homogeneous across populations. In both samples, the rs1878326-C allele tended to be more frequent in cases than in controls, 0.36 vs 0.32, *P* = 0.184 and 0.41 vs 0.35, *P* = 0.053, in French and German, respectively. The corresponding OR for AMD were then 1.19 [0.92–1.53] in French and 1.27 [1.00–1.61] in German. The two ORs were not significantly different (*P* = 0.92). Even though statistical significance was not reached separately in each sample (likely due to small sample size), the resulting combined OR for AMD associated with the rs1878326-C allele was 1.23 [1.03–1.46] and achieved significance (*P* = 0.019).

**Table 3:** Genotype distribution of MFGE8 rs4945 (R3S) polymorphism in AMD patients and controls.

MFGE8 rs4945	FCTL, n = 241	FAMD, n = 271	GCTL, n = 260	GAMD, n = 308
CC	122 (51%)	110 (41%)	118 (45%)	154 (50%)
CA	99 (41%)	128 (47%)	112 (43%)	125 (41%)
AA	20 (8%)	33 (12%)	30 (12%)	29 (9%)
MAF	0.29	0.36	0.33	0.30
OR [95%CI]	1.376 [1.056–1.791]		0.855 [0.665–1.100]	
*P*	0.017		0.229	

MAF: Minor Allele Frequency.

OR: Allelic Odds Ratio with its 95% Confidence Interval.

*P*: Cochran-Armitage trend test's p-value.

**Table 4:** Genotype distribution of MFGE8 rs1878326 (M76L) polymorphism in AMD patients and controls.

MFGE8 rs1878326	FCTL, n = 251	FAMD, n = 274	GCTL, n = 263	GAMD, n = 317
AA	108 (43%)	114 (42%)	112 (43%)	113 (35%)
AC	123 (49%)	121 (44%)	116 (44%)	148 (47%)
CC	20 (8%)	39 (14%)	35 (13%)	56 (18%)
MAF	0.32	0.36	0.35	0.41
OR [95%CI]	1.186 [0.919–1.531]		1.271 [1.001–1.613]	
*P*	0.184		0.053	

MAF: Minor Allele Frequency.

OR: Allelic Odds Ratio with its 95% Confidence Interval.

*P*: Cochran-Armitage trend test's p-value.

The pattern of linkage disequilibrium was homogeneous across populations and was characterized by a moderate linkage disequilibrium between the two studied SNP (r^2^ = 0.07, D′ = −0.53 in French and r^2^ = 0.10, D′ = −0.60) in German). As a consequence, the two SNP generated 4 common haplotypes (Table 5) among which the two haplotypes carrying the rs1878326-C allele were more frequent in cases than in controls in the German and the French study, confirming the association of the rs1878326-C allele with AMD observed in the univariate analysis.

**Table 5:** Haplotype frequency distributions derived from MFGE8 rs1878326 (M76L) and rs4945 (R3S) polymorphisms in AMD patients and controls.

Polymorphisms		French		German	
rs1878326	rs4945	FCTL (n = 238)	FAMD (n = 269)	GCTL (n = 253)	GAMD (n = 298)
A	C	0.426	0.342	0.355	0.344
A	A	0.252	0.293	0.289	0.245
C	C	0.284	0.299	0.310	0.359
C	A	0.038	0.066	0.045	0.052

### MFGE8 expressing cells in the retina

**Figure 1:**
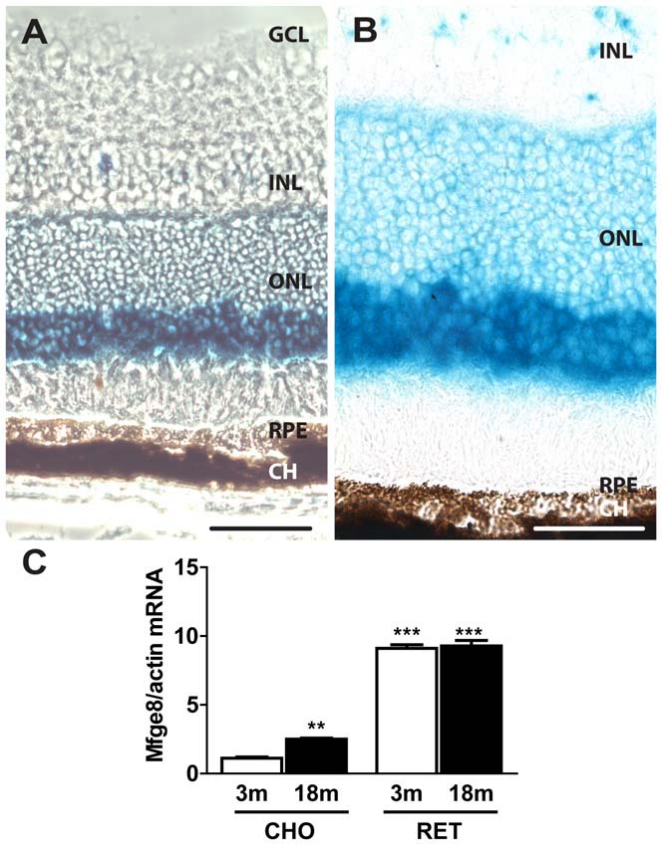
MFGE8 expressing cells in mouse retina. Representative micrographs of ß-galactosidase localization in MFGE8^+/GAL^ mice detected by a ß-galactosidase detection reagent (blue staining). Phase contrast (A) image and light transmitted (B) image. CH: choroid; GCL: ganglion cell layer; INL: inner nuclear layer; ONL: outer nuclear layer; RPE: retinal pigment epithelium. Experiments were reproduced at least 3 times. Scale bars: 100 µm (A); 25 µm (B). *Mfge8* RT PCR in neural retina (RET) and choroid (CHO) at different time points in C57Bl6 mice (3 month old vs 18 month old, n = 6/group; Dunnett test with 3 m old CHO as control, **, *P*≤0,01 and ***, *P*≤0,001).

In the litterature, immunohistochemistry using an anti-MFGE8 antibody localized MFGE8 protein mainly to the inner segments of photoreceptors and to a lesser extent to RPE cells in mice using two different antibodies [14]. We identified MFGE8 expressing cells in *Mfge8^+/−^* mice thanks to the gene-trap insertion of ß-galactosidase in the *Mfge8* gene. The resulting fusion protein is retained in the cell and ß-galactosidase activity therefore allows to identify MFGE8 expressing cells [8]. ß-galactosidase, detected by a ß-galactosidase detection reagent, mainly localizes to the outer nuclear layer (ONL) containing photoreceptor cell bodies (Fig. 1A). Higher magnification reveals that staining is particularly strong in the outer part of the ONL and that the RPE does express little ß-galactosidase (Fig. 1B), confirming the previously published results using MFGE8 antibodies [14]. There was no endogenous ß-galactosidase detection in WT mice (data not shown). We confirmed this staining pattern using RT PCR in separated tissues (Fig. 1C). *Mfge8* mRNA was highly expressed in young and aged neural retina. In the RPE/choroid, basal mRNA expression was significantly lower than in the neural retina. A slight but significant increase in *Mfge8* mRNA expression at 18 month old compared to 3-month-old C57Bl6 mice was observed. Taken together, our results identify photoreceptors as the main source of MFGE8 in the eye and suggest that MFGE8 localization in the RPE cells [14] is due to uptake rather than RPE MFGE8 expression.

### MFGE8 and retinal homeostasis

**Figure 2:**
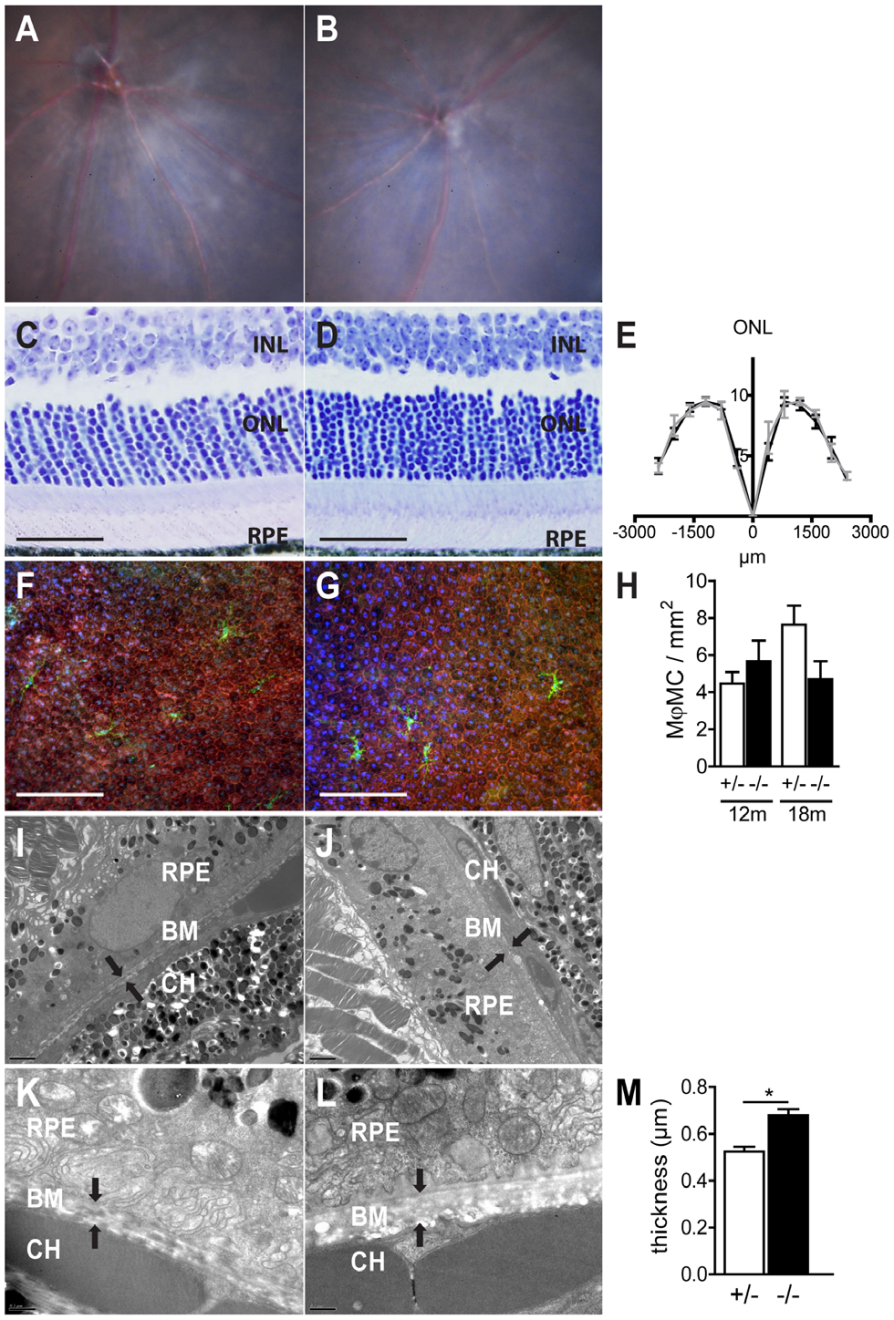
MFGE8 and retinal homeostasis. Fundus photographs of 18 month-old *Mfge8^+/−^* mice (A) and *Mfge8^−/−^* mice (B). Histological sections of historesin-embedded eyes from 18 months old *Mfge8^+/−^* mice (C) and *Mfge8^−/−^* mice (D). Quantification of the number of photoreceptor cell nuclei layers in the ONL from the inferior to the superior pole in 18 month old *Mfge8^−/−^* (E grey line) compared to *Mfge8^+/−^*(E black line, n = 4 mice/group). Rhodamine phalloidine (red) IBA1 (green) stained RPE flatmounts of 18 month-old *Mfge8^+/−^* mice (F) and *Mfge8^−/−^* mice (G). Quantification of subretinal macrophages/microglia (Mϕ/MC) IBA1 positive cells, (H, n = 6–8 mice/group). Representative transmission electron micrograph of Bruchs membrane (black arrows) of 18 month old *Mfge8^+/−^* (I and K) and *Mfge8^−/−^* (J and L) mice. Bruchs membrane thickness measurements in 18 month old *Mfge8^+/−^* and *Mfge8^−/−^* mice (M, *P*≤0,05, n = 4 mice/group) INL: inner nuclear layer; ONL: outer nuclear layer; RPE: retinal pigment epithelium; BM: Bruch membrane; CH: Choroid. Scale bars: 50 µm (C, D), 150 µm (F, G); 2 µm (I, J); 0,5 µm (K,L).

MFGE8 has been shown to be essential in diurnal OS phagocytosis [15] and disturbance of the phagocytosis of spent OS by the RPE, as observed in the RCS rat, leads to photoreceptor degeneration [10]. To evaluate if MFGE8 deficiency alters long-term retinal homeostasis, we first studied the fundoscopic appearance of 16–18 month old *Mfge8^+/−^* (Fig. 2A) and *Mfge8^−/−^* (Fig. 2B). Both fundi appeared smooth, devoid of any remarkable lesions, and similar to each other. Furthermore, histological sections of aged *Mfge8^+/−^* (Fig. 2C) and *Mfge8^−/−^* mice (Fig. 2D) showed a regular photoreceptor layer and quantification of the number of photoreceptor cell nuclei layers in the ONL revealed no signs of degeneration from the inferior to the superior pole in *Mfge8^−/−^* (Fig. 2E grey line) compared to *Mfge8^+/−^* mice (Fig. 2E black line). Labelling for RPE (phalloidine, red) and subretinal macrophages/microglial cells (IBA1, green) in aged *Mfge8^+/−^* (Fig. 2F) and *Mfge8^−/−^* mice (Fig. 2G) showed no morphological abnormalities of RPE cells and no pathological accumulation of phagocytes under the retina. Instead, we found a tendency of decreased subretinal phagocytes in 18-month-old *Mfge8^−/−^* compared to *Mfge8^+/−^* (Fig. 2H, quantification of subretinal IBA1 positive cells). Laminar deposits in the BM and Drusen in the RPE/BM complex are early signs of AMD that ultimately impact RPE and photoreceptor health [18], [32]. To evaluate if MFGE8 is implicated in homeostasis of BM, we evaluated BM morphology of 16–18 month old *Mfge8^+/−^* (Fig. 2I and K) and *Mfge8^−/−^* (Fig. 2J and L) by electron microscopy. Although retina and RPE were ultrastructurally similar, we observed a very modest, but significant thickening of BM in *Mfge8^−/−^* mice by 22% (Fig. 2M). To evaluate possible differences in genes that are potentially involved in lipid clearance from the BM, we analyzed *Abca1*, *Abca4*, *Ldlr*, *cd36* and *Caveolin1* mRNA in aged *Mfge8* ^+/−^ and *Mfge8^−/−^*. However, we were not able to detect an influence of MFGE8 on the expression of these genes (Figure S1). Since BM is rich in collagen, and MFGE8 has been shown to promote collagen phagocytosis to prevent reactive fibrosis [33], BM thickening observed in aged *Mfge8^−/−^* mice could be a result of a reactive fibrosis.

### MFGE8 and choroidal homeostasis and neovascularization

**Figure 3:**
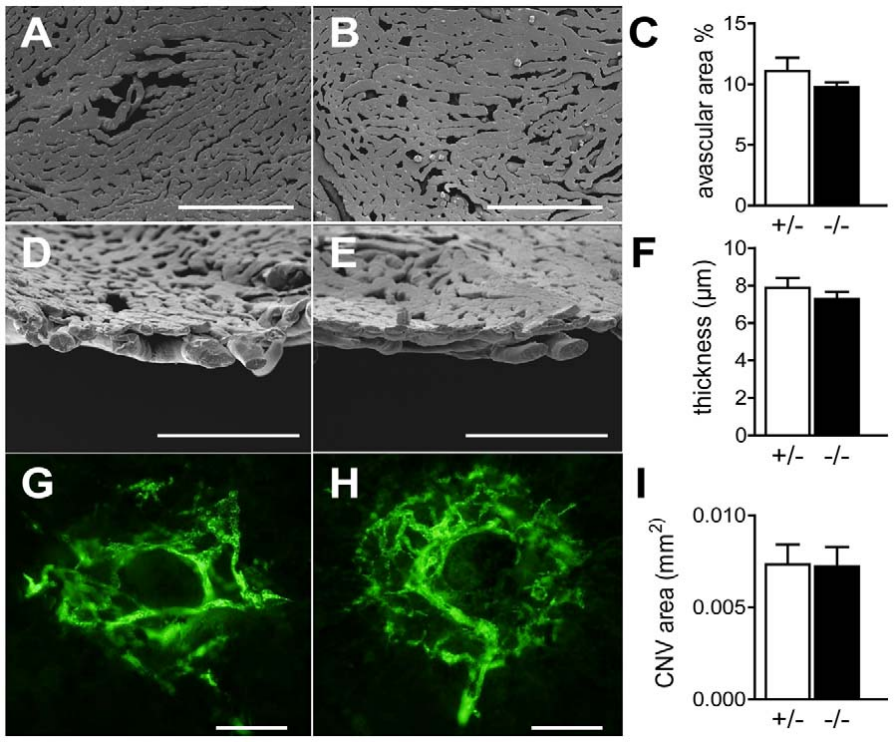
MFGE8 and choroidal homeostasis and neovascularization. Vascular corrosion casts of the retinal aspect of choriocapillaries of 16–18 month old *Mfge8^+/−^* (A) and *Mfge8^−/−^* mice (B). Quantification of the avascular intracapillary area in *Mfge8^+/−^* and *Mfge8^−/−^* mice (C, 5 mice/group). Vascular corrosion casts of perpendicularly cut choroid of 16–18 month old *Mfge8^+/−^* (D) and *Mfge8^−/−^* (E). Quantification of the thickness of the choriocapillaries in *Mfge8^+/−^* and *Mfge8^−/−^* mice (F, 3 mice/group). FITC-dextran perfused choroidal flatmounts at 14 d after laser-induced neovascularization of *Mfge8^+/−^* (G) and *Mfge8^−/−^* (H). Quantification of FITC positive choroidal neovascularizations in *Mfge8^+/−^* and *Mfge8^−/−^* mice (I, n = 8 mice/group). Scale bars: 200 µm (A, B); 100 µm (D, E); 50 µm (G, H).

Although MFGE8 deficiency did not lead to retinal degeneration, disturbances in RPE biology and diminished expression of trophic factors can lead to choroidal involution [16]. However, vascular corrosion casts of of 16–18 month old *Mfge8^+/−^* (Fig. 3 I and D) and *Mfge8^−/−^* (Fig. 3 B and E) showed no MFGE8-related vascular drop-out, measured as a percentage of intercapillary area of the total area, (Fig. 3C) or thinning, measured on pericentral, perpendicular sections through the vascular casts (Fig. 3F).

MFGE8 interacts with αvβ3 and αvβ5 on vascular endothelium and specifically promotes VEGF-driven ischemic neovascularization *in vivo*, but not bFGF induced neovascularization [8]. Since laser-induced CNV has been shown to be VEGF and integrin dependent [34], [35], we quantified CNV in the MFGE8-deficient mice. Interestingly, FITC-dextran perfused choroidal flatmounts at 14 d after laser-induced neovascularization of *Mfge8^+/−^* (Fig. 3G) and *Mfge8^−/−^* (Fig. 3H), showed no difference of CNV in the two groups (Fig. 3I). These results are consistent with our recent observation that the promoting function of MFGE8 on development of bladder tumors is not linked with alterations in intra-tumor angiogenesis [36]. Thus, the interactions of MFGE8, integrin dimers and VEGF are possibly tissue dependent.

## Discussion

MFGE8s essential role in diurnal OS phagocytosis [15] and critical involvement in VEGF-driven neovascularization [8] make it a candidate gene for an involvement in the pathogenesis of AMD, where photoreceptor degeneration, choroidal involution, or CNV can occur. We here show a suggestive statistical evidence for association of the rs1878326-C allele with increased risk of AMD in two European populations.

To evaluate the influence of MFGE8 dysfunction *in vivo* on retinal homeostasis and CNV we analyzed *Mfge8^+/−^* and *Mfge8^−/−^* mice with ageing and in a model of CNV. We first confirmed that MFGE8 is constitutively expressed by photoreceptors *in vivo*. Interestingly, the suppression of the diurnal peak of OS phagocytosis observed in *Mfge8^−/−^* mice [15] has very little negative effect on long term chorioretinal homeostasis in 18-month-old knockout mice, apart from a slight thickening of the BM. These findings confirm and extend the lack of retinal degeneration in 12-month-old *Mfge8^−/−^* mice [37]. Furthermore, even though MFGE8 interacts with αvβ3 and αvβ5 on the vascular endothelium and strongly promotes VEGF-driven neovascularization [8], it has no effect on laser-induced CNV which is VEGF and αvβ3 integrin dependent [34], [35].

Taken together, our data shows that MFGE8 is expressed in the retina but not critically involved in retinal homeostasis or CNV. It seems unlikely that it is predominantly involved in the pathogenesis of AMD. Although we present mostly negative results, we feel that this extensive *in vivo* study is of interest to researchers in the field of AMD pathogenesis, due to its exclusion of a high potential candidate gene. We hope that the publication of these negative results are helpful to the community to exclude a valid hypothesis and avoid repetetion of expensive research in other laboratories.

## Supporting Information

Figure S1 ***Abca1*** **,** ***Abca4*** **,** ***Ldlr*** **,** ***Cd36*** **,** ***Cav1*** **RT PCR in choroid/RPE in 16–18 month old** ***Mfge8^−/−^*** **(−/−) compared to age-matched** ***Mfge8^+/−^*** **(+/−).** 8 eyes/group; no statistical difference in all groups.
(TIF)
