# Supplementary figures and images for: MFGE8 Does Not Influence Chorio-Retinal Homeostasis or Choroidal Neovascularization in vivo
Source: PLoS One. 2012 Mar 15;7(3):e33244. doi: 10.1371/journal.pone.0033244 (PMC3305292; doi:10.1371/journal.pone.0033244)

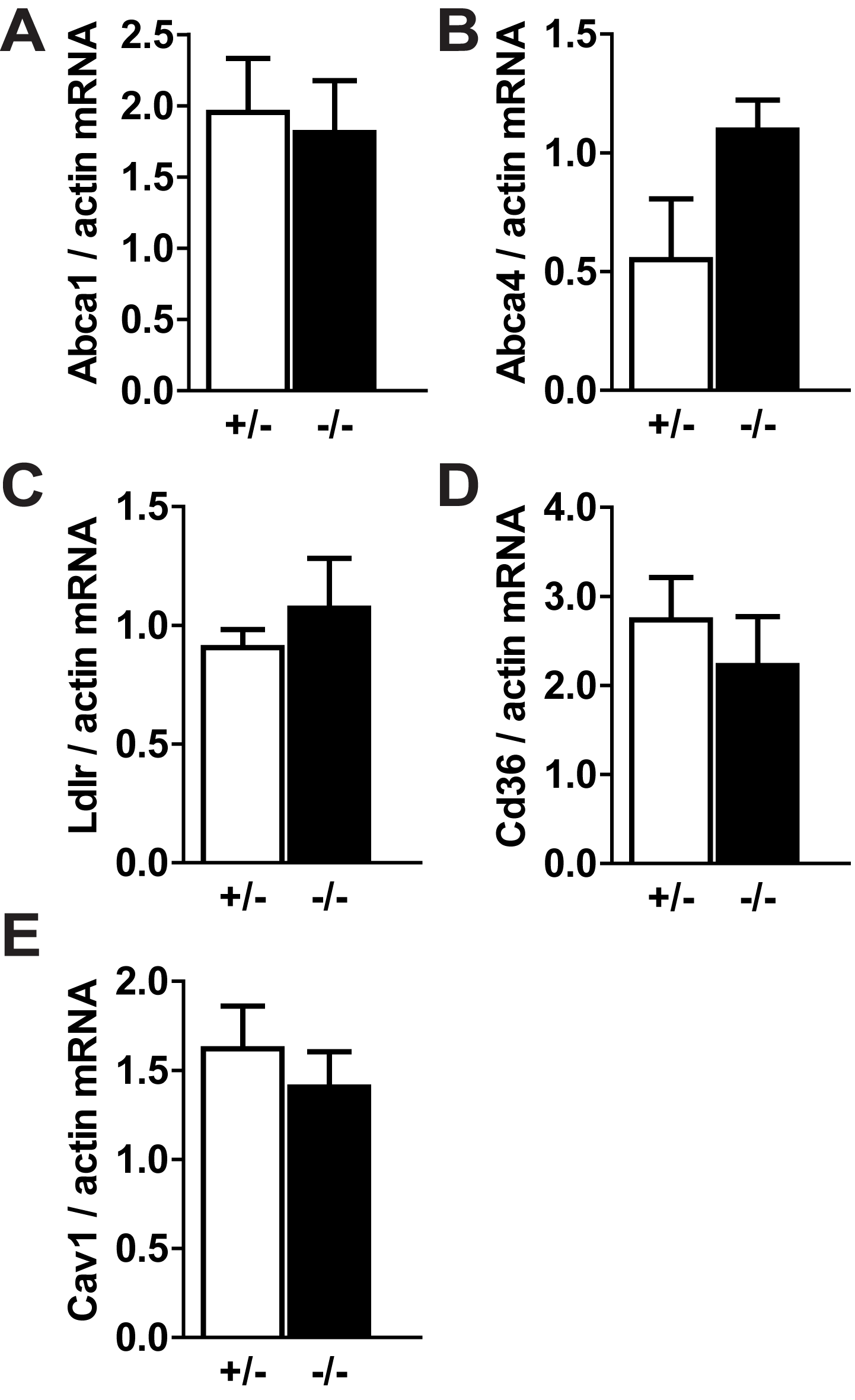

Supplement: Figure S1 — Abca1 , Abca4 , Ldlr , Cd36 , Cav1 RT PCR in choroid/RPE in 16–18 month old Mfge8−/− (−/−) compared to age-matched Mfge8+/− (+/−). 8 eyes/group; no statistical difference in all groups. (TIF) [file pone.0033244.s001.tif]
